# Notices

**Published:** 1868-02

**Authors:** 


### *Notices.*

THE BANKRUPT LAW OF THE UNITED STATES. 1867. With Notes, and a Collection of American and English Decisions upon the Principles and Practice of the Law of Bankruptcy. Adapted to the use of the Lawyer and Merchant. By Edwin James, of the New York Bar, and one of the framers of the recent English Bankruptcy Amendment Act. New York. Harper & Brothers. 1867.

The United States Bankrupt Law which has gone into effect is one so well devised for the benefit of both debtor and creditor that, unlike those which have emanated from Congress heretofore, it will endure. Therefore the members of the legal profession will study it, and business men should understand its principles and points. In order that this may be easily done, Edwin James, Esq., recently of the English Bar and one of its most accomplished members, has, in this volume, collated those authorities, American and English, which explain and elucidate the principles which regulate and control the administration of the law of bankruptcy; and by notes to the sections of the act, written in a clear and lucid style, has done much to help the reader in the applications of its provisions. The work, consisting of 325 pages, is carefully and excellently printed on good paper; and is made available for ready reference by a complete index.

